# Patterns of pharmaceutical use for immigrants to Spain and Norway: a comparative study of prescription databases in two European countries

**DOI:** 10.1186/s12939-016-0317-9

**Published:** 2016-02-24

**Authors:** Luis Andres Gimeno-Feliu, Amaia Calderón-Larrañaga, Alexandra Prados-Torres, Concha Revilla-López, Esperanza Diaz

**Affiliations:** EpiChron Research Group on Chronic Diseases, Aragon Health Sciences Institute (IACS), IIS Aragon, Miguel Servet University Hospital, Paseo Isabel La Católica 1-3, 50009 Zaragoza, Spain; San Pablo Health Centre, C/Aguadores 7, 50003 Zaragoza, Spain; Department of Medicine, Psychiatry and Dermatology, University of Zaragoza, C/Domingo Miral s/n, 50009 Zaragoza, Spain; Red de Investigación en Servicios de Salud en Enfermedades Crónicas (REDISSEC), Carlos III Health Institute, C/Sinesio Delgado 4, 28029 Madrid, Spain; Department of Microbiology, Preventive Medicine and Public Health, University of Zaragoza, C/Domingo Miral s/n, 50009 Zaragoza, Spain; Teaching Unit of Preventive Medicine and Public Health, Aragon Health Sciences Institute (IACS), Avda. San Juan Bosco 13, 50009 Zaragoza, Spain; Department of Global Public Health and Primary Care, University of Bergen, Kalfarveien 31, 5018 Bergen, Norway; Norwegian Centre for Minority Health Research, Gullhaugveien 1-3, 0484 Oslo, Norway

**Keywords:** Norway, Spain, Emigrants and Immigrants, Drug Utilization, Pharmacoepidemiology

## Abstract

**Background:**

Although equity in health care is theoretically a cornerstone in Western societies, several studies show that services do not always provide equitable care for immigrants. Differences in pharmaceutical consumption between immigrants and natives are explained by variances in *predisposing factors*, *enabling factors* and *needs* across populations, and can be used as a proxy of disparities in health care use. By comparing the relative differences in pharmacological use between natives and immigrants from the same four countries of origin living in Spain and Norway respectively, this article presents a new approach to the study of inequity in health care.

**Methods:**

All purchased drug prescriptions classified according to the Anatomical Therapeutic Chemical (ATC) system in Aragon (Spain) and Norway for a total of 5 million natives and nearly 100,000 immigrants for one calendar year were included in this cross-sectional study. Age and gender adjusted relative purchase rates for immigrants from Poland, China, Colombia and Morocco compared to native populations in each of the host countries were calculated. Direct standardisation was performed based on the 2009 population structure of the OECD countries.

**Results:**

Overall, a significantly lower proportion of immigrants in Aragon (Spain) and Norway purchased pharmacological drugs compared to natives. Patterns of use across the different immigrant groups were consistent in both host countries, despite potential disparities between the Spanish and Norwegian health care systems. Immigrants from Morocco showed the highest drug use rates in relation to natives, especially for antidepressants, “pain killers” and drugs for peptic ulcer. Immigrants from China and Poland showed the lowest use rates, while Colombians where more similar to host countries.

**Conclusions:**

The similarities found between the two European countries in relation to immigrants’ pharmaceutical use disregarding their host country emphasises the need to consider specific immigrant-related features when planning and providing healthcare services to this part of the population. These results somehow remove the focus on inequity as the main reason to explain differences in purchase between immigrants and natives.

## Background

Immigration is on the rise worldwide. Immigrants from different countries and cultures have become a natural part of cities, villages, factories, schools and other public spaces. Most European health care systems attempt to ensure universal and high quality services, being equity one of their cornerstones. But beyond the theoretical equity in access to health care, the international literature reveals important differences in real use of host health care systems depending on the patient’s immigrant status [1–7].

Pharmacological consumption can be used as a proxy of health care utilization for epidemiological purposes [8]. Comparisons of drug consumption rates between immigrants and natives have seldom been conducted. While most such studies have targeted specific drugs [9–16], to the best of our knowledge, only a few with a global pharmacological approach have been published [17–20]. These studies revealed a global pattern of lower consumption in immigrants compared with natives, although with some exceptions [9, 10, 14]. However, most of the studies relied on health surveys, which often suffer from self-selection bias, especially among immigrants [21]. Recent reviews have pointed out the need for research based on comprehensive regional pharmacological databases, which are not subject to self-selection bias and allow analyses of drug purchase both from a global perspective and for specific drugs [4–6]. Although purchase and utilization are not necessarily equivalents, for pharmacoepidemiological studies purchased drugs are considered a good proxy of drug use [22] and both terms will be used interchangeably in this paper .

**Fig. 1:**
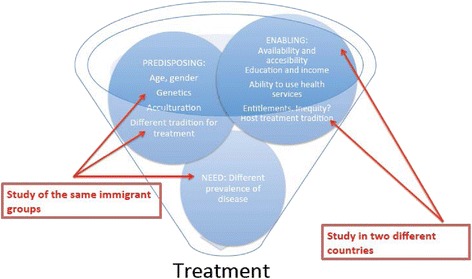
Factors influencing purchase of pharmacological treatment among immigrants. Study of four immigrant groups in two different countries. Adapted from Andersen’s health care access model, 1995

According to Andersen’s health care access model (Fig. 1), *predisposing factors*, *enabling factors*, and *need* are the three main determinants of health care use [23]. This model has previously been used for the study of utilization of prescribed drugs [8]. Among the *predisposing factors* that might explain differences in pharmacological utilization, age, sex and other demographic disparities, genetic differences, varying degrees of acculturation among immigrants and/or different cultural traditions regarding treatment inherent to country of origin have been described [13, 20, 24–26]. Besides the existence and availability of health care services, individual economic and education levels in addition to the ability to navigate through the health care services can *enable or disable* individuals to purchase prescribed medication. Last, differences in drug consumption between immigrants and natives can be appropriate if different populations have different *needs*, this is to say, different prevalence of diseases [27].

Until now, pharmacoepidemiological studies including immigrants have been carried out restricted to a host region or country. However, the host countries’ health system regulations, ways of performance, or differential local medical behaviours might also be considered as *enabling or disabling factors* for drug prescription and utilization [28, 29], resulting in different patterns of prescription for immigrants depending on which country they migrate to. For example, antidepressant consumption could be influenced by the depression prevalence in a specific immigrant group [30] but antidepressant prescription is also related to the host country’s approach regarding use of psychotherapy, duration of therapy, or even to diagnostic criteria [31]. Recently, the importance of carrying out comparative studies of immigrants in several host countries in order to clarify the influence of these factors has been underlined [1, 32].

Following this suggestion, we investigated whether patterns of use of pharmacological treatment for immigrants compared to natives were similar in two European countries: Spain and Norway. The two countries have large immigrant populations [1], and their health care systems deliver universal coverage and attempt to provide equitable health care [33]. In both countries the health systems are mainly public and rely heavily on primary health care and general practitioners (GPs). At the time of this study, immigrants and natives in Spain and Norway were equally entitled to health care services (i.e., primary care, hospital care, and emergency, public health, and pharmacy services). In the case of Spain, immigrants were entitled to these services regardless of their legal status [34], while in Norway illegal immigrants only had access to emergency health care. In Spain, users do not pay to visit the GP and those with a prescription from the National Health Service pay 40 % of the cost of acute medication and 10 % of the cost of chronic medication, with a maximum limit of €2.64 per package. Medication is free for inpatients and ‘exempt’ groups (i.e., retirees and those who have disabilities or have suffered occupational accidents) [34]. In 2010, the average copayment per patient for pharmacy medicines prescribed by the National Health Service was 5.6 % of the cost of the medication purchased [35]. In the case of private prescriptions or over-the-counter (OTC) drugs, the user must pay 100 % of the cost. It is estimated that of the total Spanish pharmaceutical market handled by the Pharmacy Service in 2010, 77.3 % corresponded to the Spanish National Health Service [35]. In Norway patients visiting their GP must pay a copayment of approximately €25 per visit. Most medications require a prescription. Patients pay for most medications for acute illnesses. Subsidised prescriptions include medications for chronic illnesses and other non-chronic diseases that require long-term treatment in a given year and only carry a nominal fee or copayment. Patient copayments currently account for 36 % of total prescription costs; the total copayment within a single calendar year is €240. Copayments for physician visits, radiology examinations, and laboratory tests are included in this amount [36]. Differences between the two countries are also observed for pharmaceutical costs as a percentage of health expenditure; this value is higher in Spain [37].

Our hypothesis was that, for immigrants moving from the same countries of origin, similar patterns of use of pharmacological treatment would be observed in Spain and Norway despite differences in these two countries regarding overall drug prescription rates. Thus, in this study we analysed all registered pharmacological treatments in Aragon (Spain) and Norway for immigrants from Poland, China, Morocco and Colombia compared to natives, aiming to identify patterns of drug use for all main and top 10 anatomical and therapeutic pharmacological groups for each immigrant group compared to host country.

## Methods

This is a cross-sectional study comparing purchase of pharmacological treatment in natives and immigrants in Spain (Aragon) and Norway. The entire population from the Aragon region (Spanish autonomous community with approximately 1.3 million inhabitants) and the whole registered population of Norway (approximately 4.8 million inhabitants, 12 % of them registered immigrants in 2008) were entitled to the respective public health systems. The period covered by the study was 1 January to 31 December 2008 for Norway and 1 January to 31 December 2010 for Aragon.

Immigrants were defined as persons whose birthplace was not the host country (Spain and Norway respectively), regardless of their nationality [38]. In order to ensure the homogeneity of study populations, only immigrants from four countries of origin were studied, each one from a different continent. From each continent we selected the country with the highest numbers of immigrants common to both host countries: Poland, China, Morocco and Colombia. Immigrant populations as a percentage of the overall populations of Aragon and Norway, respectively, were as follows: Poland, 0.3 and 0.9 %; Colombia, 0.8 and 1 %; China, 0.3 and 0.2 %; Morocco, 1.5 and 0.1 %.

Data extracted for this study both in Spain and Norway comprised all purchased drug prescriptions at first and third level of the Anatomical Therapeutic Chemical (ATC) system [39]. From the first ATC group (from now on referred to as *main ATC level*) we analysed all the groups except the “Various” groups, as this group comprises many different types of drugs, being some of them for diagnostic purposes. From the third ATC group (from now on referred to as *therapeutic ATC level*), we selected the 10 most used groups of drugs common to Norway and Aragon. Our dependent variable was the proportion of patients having purchased one or more drugs of a given ATC group at least once in the study year.

### Spanish data

The organization and management of the Spanish National Health care System are decentralized to the different autonomous regions. Aragon is an autonomous region in North-eastern Spain with 13 % of foreign nationals in 2010, a very similar proportion to the rest of the country. The population structure and the main characteristics of the Aragon Health Service are also similar to those of Spain [34]. Demographic information (age, sex and country of birth) was extracted from the central medical database of the Aragon Health Service. In order to match data from both registries, a patient identification code was used to univocally assign an anonymized number to every patient.

Data on pharmaceutical consumption was obtained from the Pharmaceutical Billing Database in Aragon. This database covers all publicly subsidised prescription drugs dispensed by pharmacies, and prescribed by public practices. Drugs prescribed in private practices or those dispensed without prescription (OTC drugs) were not included in this study, nor were drugs used in inpatient or outpatient hospital settings (i.e., very specific treatments for particular diseases and conditions). Vaccines were also excluded from this study.

### Norwegian data

Data from Norway was obtained from the National Population Register and the Norwegian Prescription Database (NorPD). The NorPD contains detailed information on all prescription drugs purchased by individual people at all pharmacies in Norway since 2004 [28]. Data are not available from either hospitals or nursing homes. Personal identification numbers assigned to all Norwegian citizens and immigrants staying in Norway for at least six months were used to link the registries together. Once obtained the identification number, the person is entitled to the same health care rights disregarding his/her immigrant background. The National Population Register provides information about all residents in Norway. Demographic information obtained from this database includes sex, age and country of origin.

### Statistical analysis

Descriptive analyses of the selected immigrant groups living in both countries were conducted. Sex and age-standardised rates of purchase of selected ATC drug groups were calculated both in Aragon and Norway for the global populations. Direct standardisation was performed based on the 2009 population structure of the OECD countries [40]. Multivariable logistic regression analyses were conducted to evaluate the purchase odds ratios (OR) and 95 % confidence intervals (CI) for several drug groups for each immigrant group relative to the native population, adjusting for age (five-year categories) and sex. The data were analysed with the STATA statistical package (version 12) and SPSS version 22; Excel 2010 (Microsoft Corporation) was used for graphical design.

The study was approved by the Clinical Research Ethics Committee of Aragon (CEICA). The Spanish part of the present work is based on the statistical analysis of anonymous data obtained with permission from the corresponding entity. The Norwegian part of this study is framed within the “Immigrants’ health in Norway” project, which was approved by the Regional Committee for Medical and Health Research Ethics and the Norwegian Data Inspectorate.

## Results

We analysed drug purchase data for around 5 million natives (Spanish and Norwegian) and for more than 96,000 immigrants. Demographic characteristics for natives and immigrants according to their country of origin are presented in Table 1. Overall, immigrants were younger than host populations in both countries; differences in sex ratios were observed depending on the immigrant’s country of origin. Higher proportions of elderly people were found among natives of both countries.

**Table 1:** Demographic characteristics of study populations

Aragon	Born in Spain	China	Colombia	Morocco	Poland
n	1,102,391	3978	10,304	18,400	3169
0–14 years %	12.4	17.3	10.6	12.9	10.2
15–64 years %	64.9	80.7	87.0	84.7	89.5
65+ years %	22.8	1.9	2.4	2.4	0.3
Mean age (SD)	44.8 (23.2)	30.7 (15.2)	33.9 (14.6)	32.5 (14.4)	33.3 (13.0)
Women %	51.1	52.9	56.7	37.2	41.5
Norway	Born in Norway	China	Colombia	Morocco	Poland
n	4,351,084	8386	4736	4824	42,787
0–14 years %	19.8	32.4	30.8	3.7	9.1
15–64 years %	63.9	62.5	68.3	91.9	89.4
65+ years %	16.4	5.1	0.8	4.4	1.4
Mean age (SD)	39.2 (23.9)	28.6 (19.8)	21.5 (12.9)	39.5 (13.6)	33.9 (13.6)
Women %	50.2	68.9	46.6	42.9	32

Table 2 shows the drug purchase rates for the main ATC groups and the 10 most frequently used therapeutic ATC groups in both host countries for the whole populations, this is to say, including natives and immigrants. Compared to Spain, a lower purchase rate was observed in Norway for all main ATC groups except for genito-urinary system and sex hormones, and for the two less used groups: antineoplastic and immunomodulating agents and antiparasitic products. Patterns were similar at the therapeutic ATC level, with Spanish rates two or three times higher for drugs for peptic ulcer and gastro-oesophageal reflux disease (18.5 % [95 % CI, 18.4–18.5] vs 5.9 % [95 % CI, 5.9–5.9]), “pain-killers” including non-steroidal anti-inflammatories and anti-rheumatic products (34.3 % [95 % CI, 34.2–34.4] vs 17.1 % [95 % CI, 17–17.1]), other analgesics and antipyretics (23.5 % [95 % CI, 23.4–23.6] vs 6.0 % [95 % CI, 6.0–6.0]), and anxiolytics (11.0 % [95 % CI, 11–11.1] vs 5.7 % [95 % CI, 5.7–5.8]). Norwegians showed a higher use of antithrombotic agents (8.7 % [95 % CI, 8.7–8.7] vs 7.1 % [95 % CI, 7.1–7.1]).

**Table 2:** Drug purchase rates in Spain and Norway adjusted by age and sex, including both immigrants and natives. All main anatomical and 10 most used therapeutic ATC groups

ATC	Drug	Spain % (95 % CI)	Norway % (95 % CI)
Main anatomical ATC groups			
A	Alimentary tract and metabolism	27.9 (27.8–27.9)	12.9 (12.9–13)
B	Blood and blood forming organs	11.4 (11.4–11.5)	10.4 (10.4–10.5)
C	Cardiovascular system	22.1 (22.0–22.2)	18.0 (17.9–18)
D	Dermatologicals	15.6 (15.5–15.7)	12.1 (12.1–12.1)
G	Genito-urinary system and sex hormones	6.8 (6.7–6.8)	14.6 (14.6–14.6)
H	Systemic hormonal preparations, excluding sex hormones and insulins	8.3 (8.3–8.4)	7.2 (7.2–7.3)
J	Antiinfectives for systemic use	32.7 (32.5–32.8)	23.7 (23.7–23.8)
L	Antineoplastic and immunomodulating agents	1.1 (1.0–1.1)	1.4 (1.4–1.41)
M	Musculo-skeletal system	38 (37.9–38.1)	18.5 (18.5–18.6)
N	Nervous system	35.2 (35.14–35.4)	24.5 (24.4–24.5)
P	Antiparasitic products, insecticides and repellents	0.8 (0.8–0.9)	1.9 (1.8–1.9)
R	Respiratory system	33.9 (33.8–34)	23.7 (23.6–23.7)
S	Sensory organs	15.4 (15.3–15.4)	12.1 (12.1–12.2)
Main therapeutic ATC groups			
A02B	Drugs for peptic ulcer and gastro-oesophageal reflux disease	18.5 (18.4–18.5)	5.9 (5.9–5.9)
B01A	Antithrombotic Agents	7.1 (7.1–7.1)	8.7 (8.7–8.7)
C10A	Lipid modifying agents	9.8 (9.7–9.8)	8.3 (8.3–8.3)
J01C	Beta-lactam antibacterials, penicillins	20.4 (20.3–20.6)	15.7 (15.7–15.8)
J01F	Macrolides, lincosamides and streptogramins	7.3 (7.3–7.4)	6.4 (6.4–6.5)
M01A	Antiinflammatory and antirheumatic products, non-steroids	34.3 (34.2–34.4)	17.1 (17–17.1)
N02B	Other analgesics and antipyretics	23.5 (23.4–23.6)	6.0 (6.0–6.0)
N05B	Anxiolytics	11.0 (11–11.1)	5.7 (5.7–5.8)
N06A	Antidepressants	7.0 (6.9–7.0)	5.9 (5.8–5.9)
R06A	Antihistamines for systemic use	11.2 (11.1–11.3)	10.7 (10.7–10.7)

**Fig. 2:**
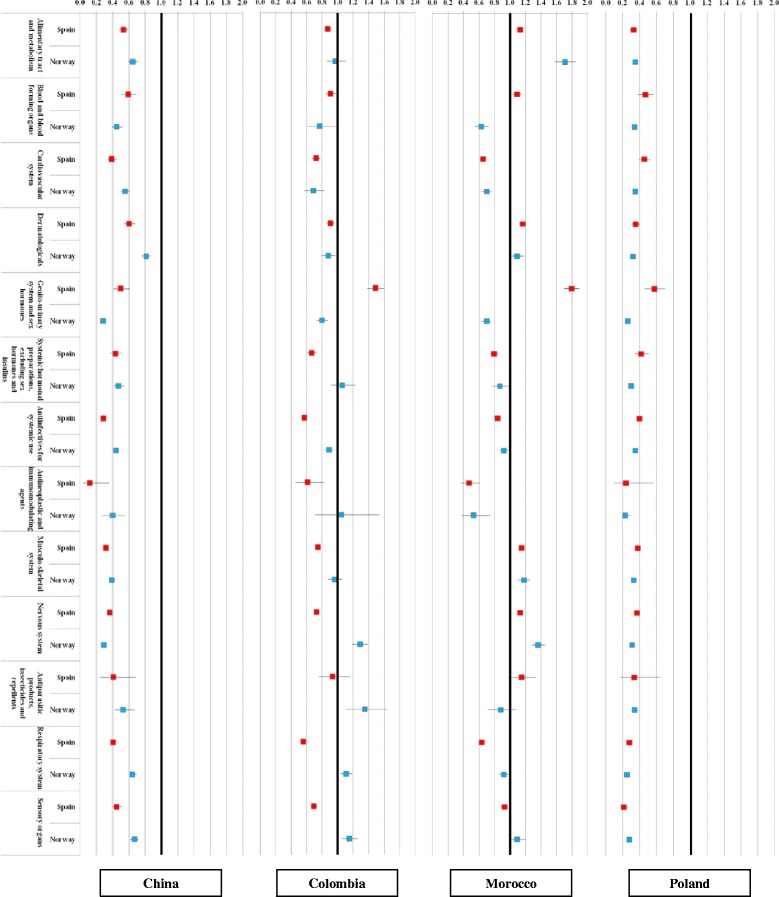
Relative drug purchase rates (main ATC groups) for immigrants from China, Colombia, Morocco and Poland in Spain and Norway compared with the native population. Odds ratios and 95 % confidence intervals adjusted by age and sex

Figure 2 depicts relative drug purchase rates between immigrants and their respective host populations at the main ATC levels for Spain and Norway. A high consistency of relative use rates across immigrants from each of the four selected countries of origin was observed in both host countries, especially so for Chinese and Polish immigrants. Immigrants from these two countries had significantly lower ratios of use of all groups of medication compared to those for natives, with most OR below 0.6 for immigrants. A significantly higher proportion of immigrants from Morocco purchased several drug groups compared to natives, particularly drugs for the alimentary tract and metabolism, dermatological, musculo-skeletal and nervous systems. However, a lower share of them purchased cardiovascular and antineoplasic medication. The principal difference in relative use between Norway and Aragon was seen for genito-urinary system and sex hormones, which was higher only in Spain. Colombians had lower use rates than natives in most drug groups, especially those living in Spain. One exception was the higher purchase rate of genito-urinary system and sex hormones drugs for Colombians and Moroccans in Spain. Table 3 includes additional data for the results shown in Fig. 2 (data are expressed as the odds ratio and 95 % confidence interval).

**Table 3:** Relative drug purchase rates (main ATC groups) for immigrants from China, Colombia, Morocco and Poland in Spain and Norway compared with the native population. Odds ratios and 95 % confidence intervals adjusted by age and sex

Drug	Host country	China	Colombia	Morocco	Poland
Alimentary tract and metabolism	Spain	0.54 (0.49–0.59)	0.87 (0.83–0.92)	1.13 (1.09–1.17)	0.33 (0.29–0.37)
	Norway	0.65 (0.59–0.71)	0.97 (0.86–1.11)	1.71 (1.58–1.84)	0.35 (0.33–0.36)
Blood and blood forming organs	Spain	0.59 (0.51–0.69)	0.91 (0.84–0.98)	1.09 (1.03–1.15)	0.47 (0.39–0.56)
	Norway	0.45 (0.39–0.52)	0.77 (0.61–0.98)	0.63 (0.55–0.72)	0.34 (0.31–0.37)
Cardiovascular system	Spain	0.39 (0.33–0.45)	0.72 (0.68–0.78)	0.65 (0.61–0.69)	0.46 (0.39–0.53)
	Norway	0.55 (0.50–0.61)	0.69 (0.57–0.83)	0.70 (0.63–0.77)	0.35 (0.33–0.37)
Dermatologicals	Spain	0.60 (0.54–0.67)	0.91 (0.86–0.96)	1.16 (1.11–1.21)	0.35 (0.3–0.41)
	Norway	0.81 (0.75–0.87)	0.88 (0.80–0.97)	1.09 (1.00–1.18)	0.32 (0.3–0.33)
Genito-urinary system and sex hormones	Spain	0.50 (0.41–0.61)	1.49 (1.38–1.60)	1.79 (1.70–1.89)	0.57 (0.46–0.71)
	Norway	0.28 (0.26–0.31)	0.80 (0.73–0.88)	0.70 (0.63–0.76)	0.26 (0.25–0.27)
Systemic hormonal preparations, excluding sex hormones and insulins	Spain	0.43 (0.37–0.51)	0.67 (0.61–0.73)	0.79 (0.75–0.85)	0.42 (0.35–0.51)
	Norway	0.47 (0.42–0.54)	1.06 (0.92–1.23)	0.87 (0.77–0.98)	0.3 (0.28–0.33)
Antiinfectives for systemic use	Spain	0.29 (0.26–0.31)	0.57 (0.55–0.60)	0.84 (0.81–0.86)	0.4 (0.36–0.43)
	Norway	0.44 (0.41–0.47)	0.89 (0.83–0.96)	0.92 (0.86–0.98)	0.35 (0.33–0.36)
Antineoplastic and immunomodulating agents	Spain	0.12 (0.04–0.36)	0.61 (0.46–0.82)	0.47 (0.37–0.61)	0.24 (0.1–0.57)
	Norway	0.40 (0.27–0.56)	1.05 (0.71–1.54)	0.53 (0.38–0.75)	0.23 (0.19–0.28)
Musculo-skeletal system	Spain	0.32 (0.29–0.35)	0.75 (0.72–0.78)	1.15 (1.11–1.18)	0.38 (0.34–0.41)
	Norway	0.39 (0.36–0.43)	0.96 (0.88–1.06)	1.18 (1.10–1.26)	0.33 (0.32–0.34)
Nervous system	Spain	0.36 (0.33–0.40)	0.73 (0.70–0.77)	1.13 (1.09–1.16)	0.37 (0.33–0.41)
	Norway	0.29 (0.27–0.32)	1.29 (1.19–1.39)	1.36 (1.28–1.45)	0.31 (0.3–0.32)
Antiparasitic products, insecticides and repellents	Spain	0.41 (0.25–0.68)	0.94 (0.76–1.16)	1.15 (0.99–1.33)	0.33 (0.17–0.64)
	Norway	0.53 (0.43–0.67)	1.35 (1.11–1.64)	0.88 (0.71–1.08)	0.34 (0.3–0.38)
Respiratory system	Spain	0.40 (0.37–0.44)	0.56 (0.53–0.59)	0.63 (0.61–0.65)	0.28 (0.25–0.31)
	Norway	0.64 (0.61–0.70)	1.11 (1.04–1.19)	0.92 (0.86–0.99)	0.25 (0.24–0.26)
Sensory organs	Spain	0.45 (0.40–0.51)	0.69 (0.65–0.74)	0.93 (0.88–0.97)	0.21 (0.17–0.26)
	Norway	0.67 (0.62–0.72)	1.15 (1.05–1.26)	1.09 (1.01–1.20)	0.28 (0.26–0.29)

**Fig. 3:**
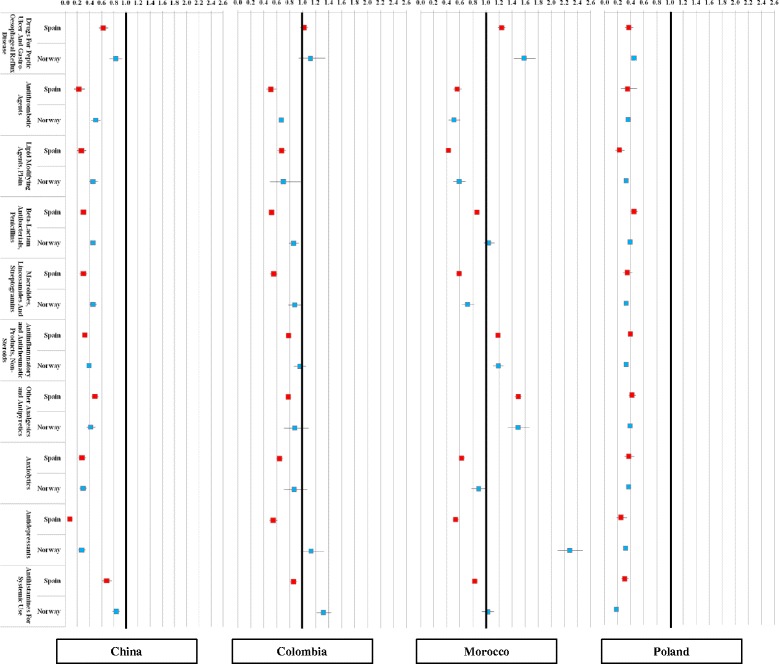
Relative drug purchase rates (ten most used therapeutic ATC groups) for immigrants from China, Colombia, Morocco and Poland in Spain and Norway compared with the native population. Odds ratios and 95 % confidence intervals adjusted by age and sex

Figure 3 presents relative drug purchase rates for immigrants compared to their host populations for the 10 most frequently used drugs at the third therapeutic ATC level. In this case, again, most immigrants’ purchase rates were significantly lower for all drug groups in Norway and Spain. Moroccans presented higher use rates for drugs for peptic ulcer and gastro-oesophageal reflux disease, non-steroids anti-inflammatory and anti-rheumatic products and other analgesics and antipyretics, and anxiolytics in both host countries, and higher antidepressant purchase rates in Norway whilst lower in Spain. Colombians presented lower or similar rates except for antihistamines for systemic use in Norway. As it happened for the main ATC levels, there was high consistency across the relative use rates of most drug groups in Norway and in Spain, especially so for Polish and Chinese immigrants. Table 4 includes additional data for the results shown in Fig. 3 (data are expressed as the odds ratio and 95 % confidence interval).

**Table 4:** Relative drug purchase rates (ten most used therapeutic ATC groups) for immigrants from China, Colombia, Morocco and Poland in Spain and Norway compared with the native population. Odds ratios and 95 % confidence intervals adjusted by age and sex

Drug	Host country	China	Colombia	Morocco	Poland
Drugs For Peptic Ulcer And Gastro-Oesophageal Reflux Disease	Spain	0.63 (0.55–0.71)	1.02 (0.96–1.09)	1.24 (1.18–1.30)	0.37 (0.32–0.44)
	Norway	0.83 (0.73–0.94)	1.12 (0.94–1.35)	1.58 (1.43–1.76)	0.45 (0.42–0.48)
Antithrombotic Agents	Spain	0.22 (0.15–0.32)	0.51 (0.44–0.60)	0.56 (0.51–0.63)	0.36 (0.25–0.50)
	Norway	0.50 (0.43–0.58)	0.67 (0.47–0.94)	0.51 (0.43–0.61)	0.36 (0.33–0.39)
Lipid Modifying Agents, Plain	Spain	0.26 (0.20–0.34)	0.67 (0.61–0.74)	0.43 (0.39–0.47)	0.23 (0.17–0.31)
	Norway	0.46 (0.40–0.54)	0.70 (0.50–0.97)	0.59 (0.50–0.69)	0.33 (0.30–0.36)
Beta-Lactam Antibacterials, Penicillins	Spain	0.30 (0.27–0.33)	0.52 (0.49–0.55)	0.87 (0.83–0.90)	0.45 (0.40–0.50)
	Norway	0.46 (0.42–0.50)	0.86 (0.79–0.94)	1.04 (0.97–1.13)	0.39 (0.38–0.41)
Macrolides, Lincosamides And Streptogramins	Spain	0.29 (0.24–0.36)	0.55 (0.50–0.61)	0.59 (0.55–0.63)	0.35 (0.28–0.43)
	Norway	0.46 (0.41–0.52)	0.88 (0.78–0.99)	0.72 (0.63–0.82)	0.33 (0.31–0.35)
Antiinflammatory and Antirheumatic Products, Non-Steroids	Spain	0.32 (0.30–0.35)	0.78 (0.75–0.82)	1.18 (1.15–1.22)	0.40 (0.36–0.44)
	Norway	0.39 (0.35–0.42)	0.96 (0.87–1.05)	1.19 (1.11–1.27)	0.33 (0.32–0.34)
Other Analgesics and Antipyretics	Spain	0.49 (0.44–0.54)	0.78 (0.74–0.82)	1.49 (1.44–1.54)	0.42 (0.37–0.48)
	Norway	0.42 (0.35–0.50)	0.88 (0.71–1.09)	1.49 (1.33–1.67)	0.39 (0.36–0.43)
Anxiolytics	Spain	0.27 (0.22–0.33)	0.64 (0.59–0.70)	0.63 (0.59–0.68)	0.38 (0.31–0.46)
	Norway	0.29 (0.24–0.35)	0.87 (0.71–1.07)	0.89 (0.78–1.01)	0.37 (0.34–0.40)
Antidepressants	Spain	0.08 (0.05–0.12)	0.55 (0.49–0.61)	0.54 (0.49–0.59)	0.25 (0.19–0.35)
	Norway	0.27 (0.22–0.33)	1.13 (0.97–1.33)	2.28 (2.10–2.48)	0.32 (0.29–0.34)
Antihistamines For Systemic Use	Spain	0.68 (0.61–0.76)	0.86 (0.81–0.92)	0.83 (0.79–0.87)	0.31 (0.26–0.37)
	Norway	0.84 (0.78–0.90)	1.32 (1.22–1.44)	1.03 (0.94–1.12)	0.18 (0.17–0.19)

## Discussion

In the two European countries studied, Spain and Norway, the proportion of immigrants that purchased pharmacological drugs was significantly lower than that of the corresponding native population. Patterns of use across the different immigrant groups were consistent in both host countries, despite potential disparities in prescription habits and other differences between the Spanish and Norwegian health care systems. Immigrants from Morocco showed the highest drug purchase rates in relation to natives, especially for antidepressants, “pain killers” and drugs for peptic ulcer. Immigrants from China and Poland showed lowest purchasing rates, while Colombians where more similar to host countries.

Although our study cannot explain the reasons for the lower use rates of the Polish and Chinese and the higher rates among Moroccans, previous studies have revealed a lower morbidity burden in patients from Asia and Eastern Europe and a worse health profile in African immigrants [27, 41, 42], and similar results have been found regarding life expectancy and mortality [43, 44]. Our results could thus be related to a better health status and consequently lower drug need in the case of Polish and Chinese immigrants and to worse health and higher purchase in the case of Moroccans. Adjusting drug purchase by morbidity burden could test this hypothesis in further studies.

Global prescription rates in Norway and Spain differed significantly. Accordingly, similar use patterns across immigrants living in both host countries cannot be completely explained by differences in *need* (prevalence of illness) between natives and immigrants. Following Andresen’s model (Fig. 1), we compared the same four immigrant groups and adjusted for age and sex in order to reduce some of the differences in *predisposing factors* affecting the purchase of medication by immigrants in Spain and Norway. However, although the genetics and traditions of the different immigrant groups are probably the same regardless of the country they move to, other factors such as acculturation may present less of a challenge in one host country compared to another (e.g., for Colombians in Spain, given their common language) [8, 11, 24, 25]. Nonetheless, the pattern observed for Colombian immigrants in Spain was no closer to the native pattern than that observed in Norway. Information regarding length of stay in the host country was unfortunately unavailable for this study, but could have helped us to determine wheter patterns changed with time since migration; several studies have demonstrated that health problems and health care utilization increase with time spent in the host country [32, 41].

As regard to *predisposing factors*, reasons for migration might be different in Moroccans and Colombians who decided to migrate to Norway, a country with a relatively high proportion of refugees, or to Spain, where most immigrants are labour ones. Our findings concerning antidepressant medication, which was significantly more often purchased among Colombians and Moroccans living in Norway compared to those living in Spain, has been previously described separately for each country [13, 14], and could reflect different reasons for migration in persons from the same country depending on the country they move to, as refugees have a higher prevalence of psychiatric illness compared to other immigrants [45].

Health care services and pharmacological treatment were largely free for immigrants who were entitled to public health care in both Spain and Norway at the time of the study. Therefore, apart for availability of services, other *enabling or disabling factors* like service’s friendly navigability and/or the ability or desirability to use the host country’s health services are probably essential factors determining the patterns of drug purchase among immigrants. Self-medication, which is extended among the Polish [46, 47], could explain their pattern of purchase in Spain and Norway if they buy medication when visiting their country on vacation [48]. Chinese people, on the other side, are known for using traditional medication [49] that might be available in the host countries and would not be registered in the ATC system. Unfortunately, we could not adjust our data for income and education levels, which might account for differences between groups to some extent.

Our analysis of four immigrant groups from different parts of the world, each with similar predisposing factors and needs, living in two European countries offering similar healthcare coverage, revealed medication purchasing profiles that were relatively consistent for each immigrant population. The purchase rates for most of these groups were lower than those of the host populations, which may be indicative of health care barriers for immigrants in both Spain and Norway. However, while the possibility of differences in health care equity cannot be ruled out, our results point to intrinsic differences between immigrant groups, suggesting that inequity is not the main driver of the observed differences in purchasing rates between immigrants and natives.

### Strenghts and limitations

This is a large population-based study including more than five millions natives and around 100,000 immigrants. Given the large sample size, most of the differences are statistically significant, although some of them are small and may not be clinically or socially relevant [50]. The information was obtained from electronic prescription databases, a source of information that has demonstrated a high value to study the epidemiology of drug use [14, 28, 50]. The use of linked registries avoids self-selection bias, which typically endangers the external validity of studies on immigrant health.

Several limitations of our approach should be noted. We did not include OTC prescriptions, which could be potentially important in the case of specific drugs (e.g., painkillers. A study by Carrasco-Garrido and coworkers found that 23.1 % of Spaniards had used OTC drugs within the previous two weeks, compared with 18.1 % of the immigrant population [17]. Inclusion of these data would only increase the differences in drug consumption between native and immigrant populations in Spain. Moreover, we analysed annual purchase rates as opposed to the annual amount of drugs. Accordingly, extra medication bought over the counter should not significantly alter our results. Drug prescriptions from private doctors are not registered in the Aragon Pharmacy Database, but are registered in Norway. One potential implication of this difference is the exclusion of the large number of prescriptions for hormonal birth control provided by private gynaecologists to Spanish natives, which may explain the higher relative rate of consumption of genito-urinary system drugs and sex hormones in Moroccan and Colombian women in Spain, but not in Norway. Because Spaniards are more likely to have private health insurance (17.4 %) than immigrants (12.6 %) [51], inclusion of this data would further widen the gap in drug consumption between immigrants and natives in Spain.

Prescriptions for patients in nursing homes were not available in the datasets analysed, which may partially explain the lower general purchase rates in Norway, where this phenomenon is more common than in Spain. However, this applies to individuals of 80 years and older, an age range that emcopasses very few immigrants. Because our study was focused on the relative differences within each host country, the impact of this potential under-registration on our overall findings is lessened.

A general problem using purchased data to assess drug use is also the fact that we do not know if and when the dispensed drugs are actually ingested by the patients [28]. However, in pharmacoepidemiological studies purchased drugs are considered a good proxy of drug use [22]. Another limitation could be the different years of data provenance: 2010 in the case of Spain and 2008 in Norway. Taking into account that our study compares the relative purchase rates between immigrants and natives within each country, this fact should not affect the final results. Last, we defined immigrants as persons born abroad (from Spain or Norway). Using other definitions, like nationality, would have changed results because of differences in nationalization laws in Spain and Norway. For example in Spain an immigrant needs to have lived at least 10 years in the country before he/she can apply for the Spanish nationality, but this period of time is much shorter if the immigrant comes from Latin American countries. On the other hand, in Norway the nationalization process can be very short in the case of refugees. Our choice sought to minimise this possible misclassification.

## Conclusions

The similarities found between the two European countries in relation to immigrants’ pharmaceutical use disregarding their host country, emphasises the need to consider specific immigrant-related features when planning and providing health care services to this part of the population.
